# The Role of Laser Modalities in Melanoma Management: Critical Analysis of Local Control and Palliative Applications

**DOI:** 10.3390/cancers18101672

**Published:** 2026-05-21

**Authors:** Francesco Russano, Luigi Dall’Olmo, Francesco Callegarin, Davide Brugnolo, Paolo Del Fiore, Giuseppe Sciacca, Rocco Caminiti, Marco Rastrelli, Simone Mocellin

**Affiliations:** 1 Soft-Tissue, Peritoneum and Melanoma Surgical Oncology Unit, Veneto Institute of Oncology IOV-IRCCS, 35128 Padua, Italy; francesco.russano@iov.veneto.it (F.R.); luigi.dallolmo@unipd.it (L.D.); paolo.delfiore@iov.veneto.it (P.D.F.); marco.rastrelli@unipd.it (M.R.); simone.mocellin@unipd.it (S.M.); 2 Department of Surgery, Oncology and Gastroenterology (DISCOG), University of Padua, 35128 Padua, Italy; davide.brugnolo@iov.veneto.it (D.B.); giuseppe.sciacca@studenti.unipd.it (G.S.); 3 Clinical Research Unit, Veneto Institute of Oncology IOV-IRCCS, 35128 Padua, Italy; 4 Casa di Cura Caminiti, 89018 Villa San Giovanni, Reggio Calabria, Italy; rocco.caminiti@icloud.com

**Keywords:** melanoma, laser therapy, Nd:YAG, CO_2_ laser, photodynamic therapy, skin cancer

## Abstract

Cutaneous melanoma is an aggressive skin cancer. While laser therapy is established for non-melanoma skin cancers, its role in melanoma remains controversial and largely unsupported by robust clinical evidence. The gold standard for melanoma management remains surgical excision, as it allows for definitive histopathological diagnosis, Breslow thickness measurement, and surgical margin assessment, which are essential for accurate staging. This narrative review analyzed preclinical and clinical studies evaluating various laser modalities, including Nd:YAG, CO_2_, pulsed dye, photodynamic therapy (PDT) and photothermal therapy (PTT), for efficacy, recurrence rates, and limitations in cutaneous melanoma management. Nd:YAG laser (1064 nm) showed potential for local control in thin stage I melanomas, reporting a low local recurrence rate of 0–0.7% and favorable 5-year survival in small, non-randomized cohorts. CO_2_ laser (10,600 nm) provides effective palliation and local control for in-transit or unresectable metastases, but local recurrence is highly variable, reaching up to 46.7%. Photodynamic therapy showed variable efficacy, although Chlorin e6 achieved complete local regression in a small series of metastases. A critical limitation of laser therapy is the irreversible destruction of tissue, which precludes these vital assessments. Therefore, laser treatment should be cautiously reserved for cases where standard surgery is not feasible, acknowledging that it may interfere with the evaluation of curative outcomes and accurate staging. Laser therapy is a valuable minimally invasive adjunct for local control in selected patients who are poor surgical candidates or require palliative care. Routine use is restricted by the lack of randomized controlled trials. Future studies should prioritize combination strategies with systemic or immunotherapeutic approaches to enhance overall outcomes.

## Simple Summary

Laser therapy, although established for common skin cancers, remains controversial for treating malignant melanoma because the procedure destroys the tissue required for doctors to properly diagnose and confirm that the entire tumor has been removed. This critical review was necessary to assess all existing research, from lab studies to clinical patient reports, on different kinds of laser treatment to clarify their effectiveness and limitations in melanoma management. Our findings show that while lasers cannot replace curative surgery, they offer a valuable, minimally invasive alternative for local control, especially for patients who are too ill for an operation or need relief from symptoms of advanced disease. The research community should now focus on conducting rigorous studies that combine these laser techniques with powerful systemic therapies, such as immunotherapies, to significantly improve long-term outcomes for patients.

## 1. Introduction

Malignant melanoma is an aggressive neoplasm arising from melanocytes, the cells responsible for melanin production. In more than 90% of cases, melanoma develops in the skin, while extracutaneous forms are considerably less common. Ultraviolet (UV) radiation represents the primary etiological factor, with additional contributions from genetic predisposition (BRAF, NRAS, KIT, CDKN2A, MC1R, BAP1) and phenotypic characteristics (fair skin, number/type of nevi, family history) [1].

In recent years, the clinical management of melanoma has undergone substantial changes. The introduction of effective adjuvant and neoadjuvant therapies has resulted in an approximately 18% reduction in mortality over a three-year period, profoundly altering the natural history of the disease.

Despite these therapeutic advances, melanoma incidence has continued to rise steadily for over seven decades, largely driven by increased exposure to natural and artificial UV radiation. Current incidence rates are estimated at approximately 25 new cases per 100,000 individuals in Europe, 30 per 100,000 in the United States, and up to 60 per 100,000 in Australia and New Zealand. Globally, melanoma accounts for about 1.7% of all cancer diagnoses and ranks as the fifth most commonly diagnosed malignancy in the United States [2].

Global melanoma mortality is characterized by significant disparities linked to socioeconomic development and therapeutic access. In highly developed regions, the introduction of over ten targeted and immunotherapy agents since 2011 has triggered a dramatic decrease in deaths. For instance, the United States has seen mortality fall by nearly 30% over the last decade, while deaths among US whites specifically declined by 6.4% annually between 2013 and 2017. Conversely, mortality remains disproportionately high in transitioning countries. While regions like Asia and Africa account for a relatively small fraction of global cases, they represent a much larger share of global deaths—21.0% and 4.7% respectively—often due to late-stage diagnoses and the prevalence of aggressive subtypes like acral lentiginous melanoma. Looking ahead, the total number of melanoma deaths is projected to rise by 68% by 2040, reaching approximately 96,000 annually. This increase is largely attributed to global population growth and the rising number of elderly individuals, who carry the highest age-specific mortality risks. Mortality trends, however, are unevenly distributed. Since 2013, a significant decline in melanoma-related deaths has been observed in high-income countries and among white populations, likely reflecting improved access to novel therapies for advanced disease. In contrast, mortality rates continue to increase in low-income countries and among non-white populations, highlighting persistent disparities in healthcare access [3,4,5].

From a pathogenetic standpoint, cutaneous melanoma results from the progressive accumulation of genetic mutations that disrupt cellular proliferation, differentiation, and apoptosis. In more than 75% of cases, these alterations are directly induced by UV radiation. Malignant transformation arises from the interaction between acquired oncogenic mutations—most commonly involving BRAF, NRAS, or KIT—germline genetic modifiers such as CDKN2A, MC1R, and BAP1, and phenotypic risk factors including fair skin, number and type of nevi, and a family history of melanoma.

Primary prevention strategies aimed at reducing UV exposure and promoting adequate photoprotection are universally endorsed by international health authorities and have demonstrated effectiveness in lowering the incidence of skin cancers.

Clinical screening allows melanoma to be detected at earlier stages, which are associated with a more favorable prognosis [6].

Therapeutic options include surgery, radiotherapy, chemotherapy, targeted therapies, immunotherapy, and intralesional treatment with oncolytic viruses.

Laser therapy for the treatment of skin tumors was first introduced in the 1960s following the invention of the ruby laser by T.H. Maiman [7]. The evolution of medical lasers over the past fifty years has transformed them from early experimental tools into essential instruments for precise, minimally invasive procedures such as Laser Interstitial Thermal Therapy (LITT). This technological journey began with ruby and CO_2_ gas lasers, though initial clinical applications were often limited by poor targeting and insufficient surgeon control over thermal effects. Today, medical practice employs four primary categories—solid-state, diode, dye, and gas lasers—which allow for the selective heating, vaporization, ablation, or coagulation of tissue depending on specific wavelength and energy parameters. Solid-state systems, such as the 1064 nm Nd:YAG laser, are particularly valued for their deep penetration into soft tissues, whereas diode lasers, often operating at 980 nm, are optimized for water-rich environments to create rapid lesions with sharp thermal gradients. By precisely managing the thermal dose, these devices can either sensitize tumor cells and disrupt the blood–brain barrier at temperatures between 43 °C and 45 °C or induce immediate protein denaturation and coagulative necrosis at temperatures exceeding 50 °C. Modern advancements have further refined these capabilities by integrating real-time MRI thermometry, granting surgeons optimal control to selectively ablate difficult-to-access lesions while sparing surrounding healthy structures [8].

Since then, its use has expanded considerably in dermatology due to their ability to selectively heat, vaporize, ablate, or coagulate tissue depending on wavelength and energy settings. Currently, four main categories of medical lasers are employed in clinical practice: solid-state, diode, dye, and gas lasers [8].

In recent years, laser therapy for non-melanoma skin cancers has gained increasing acceptance due to its ability to minimize scarring and reduce adverse effects while maintaining high levels of efficacy and safety [9]. While laser therapy for non-melanoma skin cancers (NMSC) is well-established due to the well-defined boundaries and superficial nature of these lesions, melanoma presents a different challenge. The invasive growth pattern, the tendency for subclinical extension into the dermis, and the absolute requirement for precise histopathological margin assessment differentiate melanoma from NMSC, thus limiting the direct extrapolation of laser techniques from the latter to the former. In contrast, the role of laser treatment in melanoma remains controversial and is not yet supported by robust clinical evidence. Despite technological advances, laser therapy remains largely contraindicated in melanoma by current international guidelines. In evaluating the clinical role of laser modalities, it is essential to distinguish between two distinct therapeutic intents: local control and palliative application. Local control’s approach aims for definitive tumor eradication, typically in primary cutaneous lesions where surgery is contraindicated or technically challenging. The goal is to achieve clearance comparable to surgical standards, although this is inherently limited by the lack of margin assessment. Palliative Application: This approach is reserved for patients with advanced, unresectable disease (e.g., in-transit or bulky cutaneous metastases). Here, the primary objective shifts from oncological cure to symptom management—specifically, the alleviation of pain, bleeding, ulceration, and infection—to improve the patient’s quality of life (QoL).

Laser therapy is based on the controlled delivery of electromagnetic energy to biological tissues, where light is absorbed by specific chromophores and converted into thermal, mechanical, or photochemical effects. The nature and extent of tissue damage depend primarily on wavelength, pulse duration, fluence, and the optical properties of the target tissue. In dermatologic applications, the main chromophores include melanin, hemoglobin, and water. When laser light is absorbed, energy is transformed into heat, producing temperature-dependent biological effects [10]. Mild temperature elevations may cause reversible cellular stress, whereas higher temperatures lead to protein denaturation, coagulation, or vaporization. Extremely short, high-energy pulses can also produce photomechanical effects, resulting in cellular disruption without significant thermal diffusion. In melanoma and other pigmented lesions, melanin represents a key chromophore. However, because melanin distribution within tumors is heterogeneous and lesions may extend deeply into the dermis or subcutaneous tissue, complete and selective tumor destruction is difficult to achieve. This limitation contributes to the ongoing uncertainty regarding the role of laser therapy in melanoma management [10]. Furthermore, recent bibliometric analyses underscore the dramatic shift in the research landscape, with a significant increase in publications focusing on immune checkpoint inhibitors (ICIs) and their clinical outcomes. While ICIs have transformed the management of advanced melanoma, the current research frontier is increasingly focused on identifying predictive models and optimizing combination strategies involving ICIs and other adjuvant modalities to overcome resistance and enhance anti-tumor efficacy [11].

## 2. Materials and Methods

A comprehensive review of the peer-reviewed literature, published in English and Italian and investigating laser therapy in cutaneous melanoma, was conducted using the main databases: PubMed/MEDLINE, Embase, and Scopus. The search covered literature published up to April 2026. This review was performed as a narrative review of the available literature and does not follow PRISMA guidelines.

The selection criteria for the analyzed studies were defined as follows, to ensure clinical relevance and interpretability of results:

- Eligible Study Types: Prospective and retrospective clinical studies, case series, cohort studies, and literature reviews were included. To provide a comprehensive overview, small case series and individual case reports were also included in selected sections where no higher-level evidence was available.
- Inclusion Criteria: Patients were required to have a histologically confirmed diagnosis of melanoma.

Studies had to clearly describe the clinical context of laser therapy application, including disease stage, lesion characteristics, and treatment intent (curative, palliative, or adjuvant).

Studies had to include adult patients, regardless of sex, ethnicity, or geographic origin. Articles were required to report at least one predefined outcome measure, such as objective or subjective treatment response, local control or recurrence rates, disease-free survival, overall survival, duration and completeness of follow-up, and safety data (adverse events, procedural complications, etc.).

Minimum Methodological Details: Studies were required to provide a clear description of the laser technology employed (e.g., type and wavelength), treatment parameters (energy settings, pulse duration, number of sessions), and procedural techniques. Articles lacking sufficient technical details to allow interpretation of the intervention were excluded.

Exclusion Criteria: Conference abstracts, editorials, letters to the editor, and expert opinions were excluded. Studies that did not directly evaluate laser therapy for melanoma management, focused exclusively on non-melanoma skin cancers, or did not provide sufficient data on clinical outcomes, recurrence rates, or follow-up duration were also excluded.

Only studies published in English or Italian and available in full-text format were included. No restrictions were applied with regard to year of publication, in order to capture the full historical and technological evolution of laser therapy in melanoma treatment.

## 3. Results

Several laser technologies have been investigated for the treatment of cutaneous melanoma and, more broadly, non-melanoma skin cancers. However, the available evidence remains heterogeneous and is largely limited to preclinical studies, case series, and retrospective analyses, with a notable lack of randomized controlled trials.

### 3.1. Physical Principles of Laser-Tissue Interaction

The clinical efficacy of laser therapy in melanoma management is governed by the specific physical mechanisms of laser-tissue interaction, which can be broadly categorized into thermal and photochemical processes. 

Thermal Mechanisms: These rely on the absorption of laser radiation by endogenous chromophores, primarily melanin, hemoglobin, and water. The choice of wavelength is critical; for instance, the 1064 nm wavelength of the Nd:YAG laser is chosen for its deep tissue penetration, whereas the 10,600 nm wavelength of the CO_2_ laser is highly absorbed by water, making it ideal for superficial tissue vaporization. The biological effect is strictly dependent on the fluence (dose) applied: moderate temperature rises induce reversible cellular stress or coagulation, while high fluence leads to immediate protein denaturation and explosive tissue ablation [8]. 

Photochemical Mechanisms (PDT): Unlike thermal ablation, Photodynamic Therapy (PDT) is a non-thermal process. It requires a light source with a wavelength precisely matched to the absorption peak of an exogenous photosensitizer. When the light excites the photosensitizer, it reacts with molecular oxygen within the tumor to produce reactive oxygen species (ROS). These ROS cause localized oxidative damage, leading to selective cell death and microvascular disruption without the need for extensive heating. Therefore, the relevance of light in PDT is not as a heat source, but as a specific trigger for a cytotoxic chemical reaction [10].

### 3.2. Laser Technologies (Thermal-Based Modalities)

Ruby and alexandrite lasers emit wavelengths that are strongly absorbed by melanin, making them highly effective for pigmented lesions. Their mechanism of action is primarily based on selective photothermolysis of melanin-containing cells. However, the depth of penetration is limited, and energy distribution may be heterogeneous in thick or deeply invasive tumors. This raises concerns about incomplete tumor destruction and residual viable melanoma cells in deeper dermal layers.

#### 3.2.1. Alexandrite Lasers (755 nm)

The Q-switched alexandrite laser (755 nm) has primarily been evaluated in vitro, where potentially unfavorable biological effects have been observed. Alexandrite laser irradiation induces thermal injury through selective photothermolysis of melanin-containing cells. Any observed changes in cell cycle regulators, including p16 expression, are therefore more likely secondary to heat-induced cellular stress rather than direct DNA damage. Laser irradiation was shown to significantly increase p16 gene expression in melanoma cell lines, suggesting the activation of potentially unfavorable stress-response pathways. Although p16 is classically involved in cell cycle regulation and senescence, its upregulation following thermal stress does not necessarily translate into effective tumor suppression and may reflect a complex cellular stress response. As anticipated, current data are confined to ex vivo and in vitro models, which fail to replicate the complex tumor microenvironment. However, the clinical translation of these findings is hindered by a significant lack of clinical validation that makes it impossible to draw definitive conclusions on the safety of these modalities in human subjects [12].

#### 3.2.2. Ruby Lasers (694 nm)

Historically, the pulsed ruby laser (694 nm) was one of the first technologies explored for its selective biological impact on pigmented tissues. Early experiments demonstrated that pigmented cells could be destroyed by a single exposure at low energy densities, such as 25 J/cm^2^, while non-pigmented cells remained remarkably resistant. When applied to human melanoma in situ, the laser successfully targeted the pigmented basal layer of the epithelium but often left surrounding non-pigmented supporting structures unharmed.

Despite this precision, clinical implementation faced significant hurdles due to incomplete tumor eradication. Histological studies of treated nodules frequently revealed a peripheral rim of viable tumor cells surviving at the margins of the central necrosis. This lack of total destruction typically resulted in local recurrence within 6 to 8 weeks. Interestingly, the destructive effect of the ruby laser was not fully explained by immediate physical energy transfer; the damage often extended well beyond the focal point of the beam and appeared progressively over several weeks. Researchers hypothesized that this “delayed effect” might be mediated by the formation of secondary cytotoxic enzymes or compounds within the tumor microenvironment. While these pioneering studies provided the foundation for laser oncology, the ruby laser eventually gave way to more advanced systems capable of achieving full-thickness ablation without such high risks of recurrence [13,14,15,16].

#### 3.2.3. Nd:YAG Laser (1064 nm)

The neodymium-doped yttrium aluminum garnet (Nd:YAG) laser (1064 nm) is a highly effective tool for treating non-melanoma skin cancers, such as basal and squamous cell carcinomas, due to its deep tissue penetration and ability to induce coagulative necrosis. By delivering high-energy pulses, it generates localized hyperthermia that destroys tumor cells and disrupts the surrounding microvasculature. Beyond direct ablation, Nd:YAG laser systems are frequently employed as excitation sources in specialized techniques such as PDT or Photothermal Therapy PTT due to their reliable power output and tissue penetration. Clinical studies have demonstrated that this approach often yields superior cosmetic results compared to conventional surgery, particularly for lesions on the face or nose where skin layers are thin. Despite these benefits, the use of Nd:YAG lasers in melanoma remains limited because surgical excision is required for accurate histological staging [17,18,19,20,21].

The main limitation of the Nd:YAG laser in melanoma is the inability to obtain histologic diagnosis and assess surgical margins, preventing its use as a standard treatment [22]. Consequently, its clinical application has been primarily investigated for palliative purposes in patients with unresectable lesions, distant cutaneous metastases, or in-transit metastases.

In a comprehensive review of 30 clinical studies, Mirza et al. (2017) [23] concluded that solid-state lasers are generally contraindicated for the management of malignant melanoma. This assessment is primarily based on the risk of triggering adverse biochemical pathways; for instance, irradiation with 755 nm Alexandrite lasers has been shown to induce DNA damage and elevate p16INK4a protein levels, which is highly undesirable in melanoma pathogenesis. Furthermore, research on the 694 nm Q-switched ruby laser revealed that while superficial pigmented cells are successfully ablated, deeper dermal melanocytes often persist. The uncertain behavior of these surviving cells—including the potential for repigmentation and malignant transformation—presents a significant clinical risk. While certain diode and gas lasers (such as pulsed CO_2_) are viewed more favorably for limited applications like lentigo maligna, they are constrained by a fundamental diagnostic limitation: the absence of pathological confirmation [23].

Early clinical evidence for Nd:YAG in melanoma dates back to Brunner et al. (1985) [24], who treated benign, semi-malignant, and four malignant melanoma lesions using high-power settings (50 W, 8000–12,000 J/m^2^, 5–8 s pulse duration). Histologic assessment two days post-treatment demonstrated complete healing with favorable cosmetic outcomes. The study suggested the Nd:YAG laser could effectively treat tumors with skin thickness ≤ 5 mm, given its penetration depth of up to 5 mm [24].

More robust clinical evidence comes from Moskalik and colleagues. In a study of 272 patients with stage I cutaneous melanoma, pulsed Nd:YAG laser treatment resulted in a remarkably low local recurrence rate of 0.7% and a five-year overall survival of 84.5%, with only mild and transient local side effects [25]. A subsequent study focusing on 47 facial stage I melanomas confirmed these results, reporting a five-year survival of 82.9% with no local recurrences, while also achieving favorable cosmetic outcomes [26]. These studies primarily consist of small, non-randomized single-center cohorts that are subject to selection bias, as they often enroll highly selected patients (e.g., those with thin, Stage I melanomas who are deemed poor surgical candidates). Consequently, the results may not be generalizable to the broader melanoma population, and the lack of randomized controlled trials (RCTs) prevents a direct comparison with the gold-standard surgical outcomes.

Nd:YAG laser shows potential for early-stage melanoma with thin lesions and for palliative management of unresectable metastases, but its routine use is limited, primarily due to lack of histologic control and risk of incomplete tumor eradication [27].

In summary, the clinical application of Nd:YAG laser in melanoma is defined by:

- Indications: Stage I, thin melanomas in patients who are poor surgical candidates or where surgery would be mutilating.
- Therapeutic Intent: Local tumor control.
- Level of Evidence: Low, based on small, non-randomized cohorts and retrospective studies.
- Limitations: Inability to obtain histological diagnosis, assess surgical margins, or verify Breslow thickness post-treatment.

#### 3.2.4. Carbon Dioxide (CO_2_) Lasers (10,600 nm)

The carbon dioxide (CO_2_) laser (10,600 nm) is the most extensively studied laser modality in metastatic cutaneous melanoma, particularly in cases where surgical excision is not feasible. Since the early 1990s, CO_2_ laser ablation has been used for satellite and in-transit metastases, demonstrating effective local disease control, symptom relief, and improved quality of life with generally low morbidity [28,29,30,31]. Based on the principle of selective photothermolysis, the target chromophore for the carbon dioxide laser is water. When used on human tissue, it rapidly heats intracellular water, leading to explosive vaporization of tissue and immediate ablation of the targeted area.

Retrospective analyses and case series report one-year survival rates ranging from 45% to 73% among treated patients, with adverse events generally limited to delayed wound healing or local infection. The number of treatment sessions required to achieve local disease control varied: some patients achieved adequate control after a single session, whereas others required multiple treatments, with an average of up to four sessions per patient. Reported durations of local disease control ranged from a few weeks to over six months in selected cases.

A ten-year retrospective study of 42 patients who underwent 105 CO_2_ laser treatments for cutaneous melanoma recurrences documented that 23 patients (54.8%) survived with a median follow-up of 5.4 years, whereas 19 patients (45.2%) died with a median follow-up of 0.8 years [32]. Among survivors, approximately 43% remained disease-free for more than one year, supporting the clinical efficacy of CO_2_ laser therapy in achieving local tumor control. Similar findings were reported in other series; for instance, among 16 patients with in-transit metastases, six achieved long-term remission without systemic progression, with minimal adverse effects [33,34].

Clinical evidence shows that CO_2_ laser therapy provides stable regional control with a median duration ranging from 14 weeks to 5.5 months. While approximately 41% of patients may achieve stability after just one session, the procedure is highly repeatable, with some individuals requiring up to 17 or 19 treatments to manage recurrent deposits. Although it does not fundamentally alter overall survival—with median survival times reported between 14 and 45 months due to systemic progression—it remains a vital palliative tool for maintaining quality of life by preventing the formation of painful, bleeding, or infected tumors [35,36].

Although CO_2_ laser vaporization is generally well-tolerated and efficient in an outpatient setting, its outcomes are not uniformly successful due to significant concerns regarding local recurrence. Prospective data have documented a striking failure rate, with recurrences at the treated sites observed in 46.7% of patients, specifically 7 out of 15 individuals, within nearly one month post-procedure. In these instances, the resulting tissue defects were rapidly refilled by recurrent tumors, a phenomenon that may be stimulated by high local growth factor concentrations during the healing process or the inadvertent transport of vital melanoma cells to the base of the laser crater. Furthermore, histological findings have occasionally revealed residual melanoma in areas where the surgeon visually judged the ablation to be complete, prompting a need for wider and deeper margins. Because of this high early recurrence rate, CO_2_ laser therapy is typically not recommended as a first-line treatment for limited disease, where surgical excision remains standard; instead, it is reserved as a palliative option for moderate to extensive cutaneous metastases or as a salvage therapy when surgical excision is no longer feasible [37]. While non-fractionated CO_2_ laser remains a well-established palliative approach, data on fractional CO_2_ laser are extremely limited, derived from very small cohorts, and insufficient to draw definitive conclusions regarding its clinical efficacy. It is critical to note the high heterogeneity across these retrospective series regarding laser energy settings, tumor burden, and follow-up protocols. The reported recurrence rates, ranging up to 46.7%, are likely underestimated in some cohorts due to inconsistent follow-up durations. The variability in these outcomes makes it difficult to ascertain the true palliative efficacy of CO_2_ laser ablation, and the retrospective nature of these studies limits our ability to assess long-term survival benefits independently of systemic therapies.

In summary, the use of CO_2_ lasers follows these parameters:

- Indications: In-transit, satellite, or unresectable cutaneous metastases.
- Therapeutic Intent: Palliation, symptom relief (e.g., bleeding, pain), and quality-of-life improvement.
- Level of Evidence: Moderate, derived from retrospective analyses and case series.
- Limitations: High local recurrence rates (up to 46.7%) and lack of impact on systemic disease progression.

#### 3.2.5. Pulsed Dye Lasers (PDL) (585–595 nm)

Several studies have investigated the integration of systemic or topical therapies with laser treatments. For instance, Chen et al. evaluated in situ immunotherapy using dinitrophenyl (DNP) hapten in combination with laser therapy in 72 patients with advanced melanoma. Dinitrophenyl (DNP) is a classic hapten used to induce contact-related delayed-type, T cell-mediated immune response (DTH), due to its potent antigenicity and high absorption in healthy skin. Laser-induced thermal injury promotes the release of tumor-associated antigens, which can then be processed and presented by antigen-presenting cells, potentially enhancing systemic antitumor immune activation. The study demonstrated that adding laser therapy enhanced immune response markers and improved survival outcomes. Specifically, the group receiving combined treatment showed a three-year overall survival rate of 25.9% and a median survival of 28 months, compared to 12.2% and 19 months in patients treated with DNP alone. Moreover, the combination therapy led to a one-year disease-free survival rate of 69.1%, significantly higher than the 44.0% observed with monotherapy. The treatment was generally well-tolerated, with mild side effects such as low-grade fever and fatigue. These results suggest that laser therapy combined with DNP may represent a promising strategy to enhance systemic immunity and reduce metastatic burden in melanoma patients. The promising survival outcomes must be viewed within the context of anecdotal evidence. These studies are typically case reports or preliminary analyses involving limited patient numbers, which introduce a high risk of reporting bias. Furthermore, the integration of topical immune modifiers (such as imiquimod) with laser therapy complicates the attribution of clinical efficacy, as it is often unclear whether the tumor regression is due to the laser-induced thermal effects or the local immunomodulation. Robust, prospective data are currently absent [38].

The clinical application of the pulsed dye laser (PDL), typically at wavelengths of 585–595 nm, is a localized, low-toxicity strategy for managing cutaneous melanoma metastases. Primarily used in palliative settings for patients who are poor candidates for surgery, the PDL works through selective photothermolysis to destroy tumor cells and induce inflammation that may trigger a localized immune response. This treatment is frequently combined with topical imiquimod 5% cream, which stimulates innate immunity via cytokine release. Case reports have shown that this synergy can lead to complete clinical resolution of widespread skin nodules. Larger retrospective reviews, such as a Mayo Clinic series, report mixed oncological outcomes: while some patients achieve durable local clearance, others experience local control alongside continued systemic progression. Ultimately, while the PDL has not demonstrated a clear ability to halt systemic disease, its primary value lies in improving quality of life and reducing the morbidity of bulky, symptomatic skin lesions [39,40].

In summary, the clinical application of Pulsed Dye Lasers (PDL) in melanoma is defined by:

- Indications: Symptomatic or widespread cutaneous melanoma metastases in palliative settings, particularly for patients who are poor surgical candidates.
- Therapeutic Intent: Localized, low-toxicity symptom control, quality-of-life improvement, and potential induction of a localized immune response (frequently when combined with topical imiquimod).
- Level of Evidence: Low/Very low, predominantly based on case reports, small case series, and limited retrospective reviews.
- Limitations: Mixed oncological outcomes, variable duration of local clearance, and a lack of demonstrated capability to halt systemic disease progression.

#### 3.2.6. Near-Infrared Diode Lasers

The experimental use of 805 nm near-infrared diode lasers is a key component of in situ photoimmunotherapy (ISPI), a novel approach that combines local photothermal destruction with immunological stimulation via topical imiquimod. By utilizing an energy-absorbing dye like indocyanine green (ICG) for amelanotic lesions, the laser induces a form of “in situ autovaccination,” where the heat-induced release of tumor antigens and heat shock proteins triggers a systemic immune response. Clinical studies have demonstrated promising efficacy in late-stage melanoma patients, including an abscopal effect where untreated regional lesions regressed following local laser treatment. Despite the advanced stage of the disease, one study reported a 12-month overall survival probability of 70% and several instances of complete clinical clearance. While local adverse events like rash (90.9%) and pruritus (81.8%) are highly frequent, the procedure is generally well-tolerated and manageable. This makes ISPI a viable palliative alternative to more toxic treatments like systemic chemotherapy or isolated limb perfusion, particularly for elderly patients or those with significant comorbidities [41,42]. Furthermore, diode lasers are increasingly favored for targeted therapies, serving as the primary light sources for both PDT and emerging nanoparticle-mediated photothermal strategies.

In summary, the clinical application of Near-Infrared Diode Lasers in melanoma is defined by:

- Indications: Late-stage melanoma and symptomatic cutaneous metastases in advanced palliative settings, serving as an alternative to more toxic treatments for elderly patients or those with significant comorbidities.
- Therapeutic Intent: Local symptom control, quality-of-life maintenance, and systemic immune stimulation (e.g., inducing an abscopal effect via ISPI combined with ICG and topical imiquimod). They also serve as light sources for PDT and nanoparticle-mediated photothermal strategies.
- Level of Evidence: Low/Very low, restricted to case reports, small experimental cohorts, and preliminary analyses.
- Limitations: High frequency of local adverse events during combination protocols (e.g., rash and pruritus) and variable long-term oncological control against systemic progression.

### 3.3. Specialized Laser-Based Techniques

#### 3.3.1. Photodynamic Therapy (PDT)

Photodynamic therapy (PDT) employs photosensitizers such as aminolevulinic acid (ALA), hematoporphyrin derivatives (HPD), chlorin e6, and methylene blue (MB), which are absorbed by metabolically active tissues and induce apoptosis via reactive oxygen species. In addition, In PDT, the light source acts as the essential catalyst for the photochemical reaction. The choice of wavelength is dictated by the depth of the target lesion and the absorption characteristics of the photosensitizer used. Unlike purely thermal laser applications, PDT is a photochemical technique that requires a light source precisely matched to the absorption peak of a specific photosensitizer. In clinical practice, this is typically achieved using diode lasers or Nd:YAG-based lasers. These devices provide the high-intensity, monochromatic radiation necessary to excite the photosensitizer and generate reactive oxygen species without inducing primary thermal damage. Case reports and series demonstrate variable efficacy:

Clinical outcomes for HPD-based photodynamic therapy in melanoma are often inconsistent, as therapeutic efficacy is significantly hindered by high tumor pigmentation. Even when local regression is achieved, systemic disease often continues to progress, with new metastases appearing as rapidly as treated lesions resolve. The clinical utility of this modality is further limited by a demanding side-effect profile, including reversible leukocytosis, elevated liver enzymes, and occasional infusion-related tachyarrhythmia. Locally, the drug’s retention in healthy skin for up to 30 days causes prolonged photosensitivity, requiring strict avoidance of sunlight. Additionally, treatment sites frequently suffer from severe pain, potential infection, and the formation of painful eschars that may take months to heal. Due to these risks and uncertain results, HPD-based therapy is generally restricted to highly selected palliative cases where conventional treatments have failed [43,44,45,46,47].

Topical PDT using 5-aminolevulinic acid (5-ALA) has been evaluated as a less invasive alternative for treating various skin malignancies, offering the advantage of restricted photosensitivity that typically resolves within 24 h. While the procedure is generally well-tolerated and requires no local anesthesia, its application in malignant melanoma has largely resulted in therapeutic failure. In clinical assessments of cutaneous melanoma metastases, 5-ALA-based PDT was found to be completely ineffective for melanotic lesions. Even in amelanotic metastases, histological analysis revealed only superficial tumor necrosis, failing to achieve deeper tumor eradication. This lack of clinical response is attributed to several physiological barriers: the overlying normal epidermis often prevents the penetration of 5-ALA into the tumor, thereby inhibiting the production of necessary photosensitizing porphyrins. Furthermore, in pigmented lesions, the melanin itself acts as an optical barrier, blocking the light penetration required to trigger a cytotoxic response. Consequently, while 5-ALA-PDT is effective for superficial epithelial tumors, it does not currently offer a viable therapeutic benefit for melanoma [48].

PDT utilizing chlorin e6 achieved a 100% complete regression rate for treated pigmented melanoma skin metastases in a clinical study of 14 patients. While eight patients required only a single course to reach total regression, the remaining six achieved full clearance after multiple sessions. Follow-up assessments ranging from 6 to 24 months showed no local recurrences in the treated areas. Despite these successful local outcomes, the long-term prognosis remained limited by systemic progression; 11 of the 14 patients eventually succumbed to distant visceral metastases, resulting in a median overall survival of 883 days. The treatment was well-tolerated with an excellent safety profile, showing no evidence of renal or hepatic injury, no changes in blood cell counts, and no cases of photodermatitis. Reported side effects were manageable, consisting primarily of localized pain lasting 2 to 5 days post-treatment and occasional transient fever or rigor [49]. Although Chlorin e6-based PDT shows potential for achieving complete local regression, the wide variability in photosensitizers and light-source parameters—ranging from aminolevulinic acid to methylene blue—results in significant methodological heterogeneity. This inconsistency makes it impossible to define a standardized PDT protocol. Furthermore, most of these studies lack a control group and suffer from a short follow-up period, which is insufficient to determine the long-term risk of systemic progression, which remains the primary cause of melanoma-related mortality in these patients.

Methylene Blue (MB)-based photodynamic therapy has been explored as an inexpensive and non-toxic alternative for treating relatively large melanoma masses in patients who are not candidates for surgery. This approach leverages MB’s high affinity for melanin and mitochondria, as well as its intense absorption in the red spectral region (600–750 nm), which facilitates a maximum light penetration depth of approximately 10 mm in living tissues. In a documented case of a 92-year-old patient, an intratumoral injection of a 2% MB solution was combined with irradiation from a halogen light source. This protocol achieved a complete clinical response in five out of six treated lesions and a partial response in the remaining site. Following several sessions, clinical tumor remission was observed over months of follow-up, notably leaving minimal to no residual scarring [50].

In summary, PDT and experimental lasers (PDL/Diode) are characterized by:

- Indications: Symptomatic cutaneous metastases in advanced palliative settings.
- Therapeutic Intent: Local symptom control and mass reduction.
- Level of Evidence: Very low, limited to case reports and small experimental series.
- Limitations: Variable clinical efficacy, high recurrence, and risk of prolonged photosensitivity.

The main outcomes and key limitations of the different types of lasers are summarized in Table 1.

#### 3.3.2. Photothermal Therapy (PTT)

An emerging experimental technique in the landscape of thermal-based treatments is Photothermal Therapy (PTT). Unlike traditional laser ablation, PTT utilizes exogenous photothermal agents, typically gold nanoparticles (AuNPs), to mediate the delivery of thermal energy to the tumor. The core of PTT lies in matching the laser wavelength with the Surface Plasmon Resonance (SPR) of the nanoparticles. When irradiated, these NPs efficiently convert light into heat, allowing for highly selective thermal destruction of malignant cells while sparing the surrounding healthy tissue. This selectivity is often enhanced by functionalizing the NPs to specifically target tumor antigens or by direct intratumoral injection [51]. Most current research employs diode lasers operating in the near-infrared (NIR) spectrum (the ‘biological therapeutic window’), where tissue absorption and scattering are minimized, allowing for deeper penetration. Furthermore, Lopes et al. (2025) [52] successfully evaluated gold nanoparticles coated with a mixture of hyaluronic and oleic acids (HAOA-AuNPs) under NIR laser irradiation; their combination therapy induced S-phase cell cycle arrest and late apoptosis in melanoma cell lines in vitro, while an in vivo proof-of-concept confirmed that intratumoral administration followed by laser scanning significantly impaired melanoma progression. Concurrently, to mitigate the risk of damaging adjacent healthy skin, research has turned toward active molecular targeting. Mikhailova et al. (2025) [53] leveraged the overexpression of the melanocortin 1 receptor (MC1R) in melanoma cells by functionalizing plasmonic gold nanorods with specific α-melanocyte-stimulating hormone (α MSH) peptides. When exposed to pulsed NIR laser regimes, these targeted agents achieved a remarkable 17.6-fold inhibition of tumor growth compared to controls, proving that hyperthermia can be precisely restricted to the malignant architecture. Green-spectrum lasers are also utilized when NPs are designed with matching SPR peaks. While continuous wave emission is standard, studies have also explored pulsed systems, such as the Nd:YAG laser, to achieve rapid thermal gradients. Despite its high precision in preclinical models, PTT is not yet clinically approved for melanoma management. Key hurdles include the long-term safety and clearance of nanoparticles from the body, the heterogeneity of NP distribution within bulky tumors, and the lack of standardized clinical protocols. Nonetheless, PTT represents a significant area of research that could eventually offer a truly selective thermal alternative to standard excision [51].

**Figure 1:**
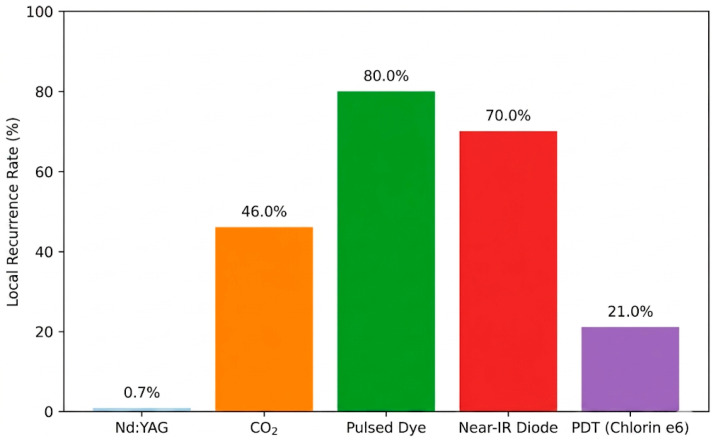
Local Recurrence rates by laser modality in clinical studies.

Figure 1 illustrates the reported local recurrence rates for different laser modalities in clinical studies. Nd:YAG laser therapy in early-stage thin melanomas is associated with the lowest recurrence, whereas CO_2_ laser therapy, mainly applied in palliative settings for metastatic or unresectable lesions, shows higher recurrence rates. Pulsed dye and near-infrared diode lasers have even higher local recurrence, reflecting limited efficacy and anecdotal evidence. Photodynamic therapy with Chlorin e6 shows intermediate recurrence, demonstrating some local control but limited systemic benefit.

**Table 1:** Comparative overview of clinical studies evaluating laser therapy in cutaneous melanoma.

Laser Type	Evidence	Clinical Setting	N° of Patients	Main Outcomes	Recurrence	Limitations
Nd:YAG (1064 nm)	Case series, retrospective	Stage I, thin melanoma	47–272	5-year OS 82–84.5%; local recurrence 0–0.7%; good cosmetic outcome	Very low	No histologic margin control; small cohorts; non-randomized
CO_2_ (10,600 nm)	Retrospective, case series	In-transit metastases	16–42	1-year survival 45–73%; effective local control; symptom relief	Variable (up to 46%)	Palliative intent; systemic progression common
Ruby (694 nm), Alexandrite (755 nm)	Ex vivo, in vitro	Ex vivo melanocytic lesions	-	Selective melanocyte damage	-	Incomplete tumor destruction; no clinical data
Alexandrite (755 nm)	Preclinical	In vitro melanoma cell lines	-	Increased p16 expression	-	Potential activation of oncogenic pathways
Pulsed Dye (585–595 nm)	Case reports	Cutaneous metastases		Local symptom relief, quality-of-life improvement	High	Limited systemic control
Near-IR diode laser (for ISPI)	Case reports	Advanced melanoma	<10	Partial/complete local response in selected cases	High	Frequent adverse events; low survival
Photodynamic Therapy (ALA, HPD, Chlorin e6, MB)	Case series	Cutaneous metastases	14	Variable efficacy; Chlorin e6 shows complete local response	High	Local control only; distant progression

## 4. Discussion

A fundamental challenge in the clinical adoption of laser therapy is the central role of histopathological parameters in melanoma management. Parameters such as Breslow thickness, presence of ulceration, and surgical margin status are the cornerstones of tumor staging and dictate the subsequent therapeutic strategy, including the need for sentinel lymph node biopsy or adjuvant therapy. Because laser modalities—such as Nd:YAG and CO_2_—induce immediate tissue destruction through necrosis or vaporization, they inherently interfere with accurate staging. This limitation represents a significant clinical risk, as the inability to histologically verify tumor depth or the completeness of eradication can lead to under-staging and potentially compromised long-term outcomes. Consequently, the use of lasers must be framed within a context of clinical necessity rather than a replacement for the surgical gold standard. Despite this overarching diagnostic limitation, this review suggests that certain modalities offer distinct benefits in highly selected contexts. Nd:YAG laser therapy, has demonstrated remarkably low local recurrence rates (0–0.7%) and favorable five-year overall survival (82–84.5%) in non-randomized studies of thin, early-stage melanomas, positioning it as a promising, minimally invasive option for patients who are poor surgical candidates. The role of laser therapy in palliative care represents a significant, yet often overlooked, facet of melanoma management. For patients with extensive or unresectable disease, the development of painful, malodorous, or bleeding cutaneous metastases significantly compromises quality of life. Laser ablation, particularly with CO_2_ lasers, provides a rapid and predictable method to debulk these symptomatic lesions. Unlike systemic therapies, which may take weeks to induce a response, laser-induced tissue vaporization offers immediate physical relief from the tumor burden. This rapid symptom control is vital for patients with short life expectancy, as it reduces the morbidity associated with ulcerated deposits and avoids the need for repetitive, potentially toxic systemic interventions. By focusing on local symptom relief and quality-of-life maintenance, laser therapy functions as a supportive care tool that addresses the immediate suffering of patients with advanced disease, filling a therapeutic gap where surgery and systemic treatments are no longer viable or desirable. Preclinical systems like the Ruby and Alexandrite lasers are largely constrained by concerns over incomplete tumor destruction and the potential activation of adverse cellular pathways, such as elevated p16 expression, mandating caution in their clinical translation. More encouragingly, the results from combination strategies, including Pulsed Dye and Near-IR Diode lasers integrated with immunotherapies (like DNP hapten or topical imiquimod) or advanced Photodynamic Therapy using Chlorin e6, suggest a synergistic potential to enhance local clearance, induce systemic anti-tumor immunity, and improve quality of life, even in advanced disease. However, it is critical to emphasize that the gold standard for melanoma management remains surgical excision, which allows for precise histopathological diagnosis, measurement of Breslow thickness, and margin assessment. Because laser therapy relies on tissue destruction (ablation or necrosis), it precludes accurate staging and the evaluation of curative outcomes. Therefore, these modalities should not be viewed as alternatives to surgery but as salvage or palliative options for cases where standard management is not feasible. On the other side we must look for new perspectives in laser management, a promising frontier in melanoma management is the integration of laser therapy with immunotherapy, targeted therapy, or local immunomodulatory treatments. The biological rationale for such combinations lies in the laser’s ability to induce thermal injury, which promotes the release of tumor-associated antigens and heat shock proteins into the tumor microenvironment. This process can act as an ‘in situ autovaccination,’ potentially enhancing the systemic anti-tumor immune response. Combining laser therapy with dinitrophenyl (DNP) or the combination of Pulsed Dye Laser (PDL) or near-infrared diode lasers with topical imiquimod 5% has demonstrated the potential for complete clinical resolution of widespread skin nodules and even untreated regional lesions through the abscopal effect. However, despite this synergistic potential, it must be clearly acknowledged that current evidence is largely derived from small-scale retrospective studies and individual case reports. The high heterogeneity of treatment protocols and the limited number of patients involved restrict the generalizability of these results. Therefore, well-designed prospective, multicenter randomized controlled trials are still required to validate the safety, standardized parameters, and long-term efficacy of these combination approaches within the multimodal melanoma strategy.

## 5. Conclusions

Laser therapy for cutaneous melanoma, while not a replacement for surgical or systemic treatments, represents a valuable adjunct in selected clinical scenarios. Nd:YAG lasers have demonstrated efficacy for thin, early-stage lesions, providing local tumor control with favorable cosmetic outcomes, whereas CO_2_ lasers are particularly useful for palliation and management of in-transit or unresectable metastases. Other laser modalities and photodynamic therapies remain largely experimental, with limited clinical evidence and variable outcomes. The primary advantage of laser interventions lies in their minimally invasive nature and ability to achieve local control with low morbidity, making them suitable for patients who are poor surgical candidates or require palliative care. However, the lack of randomized trials, heterogeneous protocols, and limited patient cohorts currently restrict their routine use. Future studies should focus on well-designed clinical trials and combination strategies with systemic or immunotherapeutic approaches, which may enhance both local and systemic tumor control. In carefully selected cases, laser therapy offers a complementary tool that can improve quality of life and expand treatment options, underscoring its potential role within a multimodal melanoma management strategy. While combination strategies involving laser therapy and immunomodulators show potential for enhancing both local and systemic tumor control, they remain in the experimental phase. Future research must prioritize rigorous clinical trials to move beyond current retrospective evidence and establish the definitive role of laser therapy in the era of systemic and targeted therapies. In conclusion, while laser therapy is a valuable tool for palliation and local control in selected patients, it remains a secondary option due to its incompatibility with standard staging protocols. The destruction of the tumor architecture prevents the assessment of Breslow thickness and margin control, which are non-negotiable for curative-intent treatment. Future clinical applications must prioritize strategies that do not compromise the accuracy of histopathological staging, ensuring that patient safety and standard oncological principles remain at the forefront of melanoma care.

## Data Availability

No new data were created or analyzed in this study.

## Abbreviations

The following abbreviations are used in this manuscript:

UV	Ultraviolet
LITT	Laser Interstitial Thermal Therapy
Nd:YAG	Neodymium-doped Yttrium Aluminum Garnet
CO_2_	Carbon Dioxide
MRI	Magnetic Resonance Imaging
OS	Overall Survival
PDL	Pulsed Dye Laser
DNP	Dinitrophenyl
DTH	Delayed-type Hypersensitivity
ISPI	In Situ Photoimmunotherapy
ICG	Indocyanine Green
PDT	Photodynamic Therapy
ALA	Aminolevulinic Acid
HPD	Hematoporphyrin Derivatives
MB	Methylene Blue
5-ALA	5-aminolevulinic acid
PTT	Photothermal Therapy
